# Unlocking Neurocognitive Substrates of Late-Life Affective Symptoms Using the Research Domain Criteria: Worry Is an Essential Dimension

**DOI:** 10.3389/fnagi.2017.00380

**Published:** 2017-11-21

**Authors:** Sherry A. Beaudreau, Nathan C. Hantke, Nehjla Mashal, Christine E. Gould, Victor W. Henderson, Ruth O'Hara

**Affiliations:** ^1^Department of Psychiatry and Behavioral Sciences, Stanford University School of Medicine, Stanford, CA, United States; ^2^Sierra Pacific Mental Illness Research Education and Clinical Centers, VA Palo Alto Health Care System, Palo Alto, CA, United States; ^3^School of Psychology, University of Queensland, Brisbane, QLD, Australia; ^4^Palo Alto Geriatric Research, Education, and Clinical Center, VA Palo Alto Health Care System, Palo Alto, CA, United States; ^5^Department of Health Research and Policy (Epidemiology), Stanford University School of Medicine, Stanford, CA, United States; ^6^Department of Neurology and Neurological Sciences, Stanford University School of Medicine, Stanford, CA, United States

**Keywords:** anxiety, cognition, cognitive control, depression, older adults, worry

## Abstract

While investigations have sought to identify the distinct and shared contributions of anxiety and depression to neurocognitive processes in late life, less is known regarding the further contribution of worry, a unique and critical dimension of affective dysregulation. Capturing the full range of symptoms, as inspired by the NIH Research Domain Criteria (RDoC), may provide finer-grained information on inter-relationships among worry, anxiety and depression on neurocognitive processing in later life. The objective of this study was to determine if the dimensional trait of worry intensifies known negative associations of dimensional measures of anxiety and depressive symptoms with neurocognitive processes, specifically cognitive control and memory processes. Using a cross-sectional and observational design, this study was conducted within a translational research center located with a Veterans medical center in Northern California. One hundred and nineteen community-residing older adults ages 65–91 years participated, and were characterized with psychiatric and neurocognitive dimensional measures. Affective symptom severity was assessed with the Penn State Worry Questionnaire, the Beck Anxiety Inventory (BAI), and the Beck Depression Inventory-II. Primary neurocognitive outcomes were inhibitory control assessed using a Stroop paradigm and delayed verbal memory assessed with the Rey Auditory Verbal Learning Test. Secondary outcomes included other less frequently examined cognitive control mechanisms (working memory, information processing, and verbal fluency) and memory processes (visual delayed memory). Contrary to prediction, the dimensional trait of worry attenuated negative associations between anxiety and depressive symptoms and inhibitory control on the one hand, and between depressive symptoms and delayed verbal memory processes on the other. In the secondary models, symptom dimensions were not associated with other cognitive control or visual delayed memory processes. Our fine-grained approach, in line with the NIMH RDoC model, suggests the neurocognitive processes associated with dimensional measures of late-life affective symptoms are dissociable. Specifically, dimensional measures of worry operate independently from other anxiety and depression symptoms to reveal differential patterns of neurocognitive processes associated with affective dysregulation.

## Introduction

Perseverative negative thought in the form of worry represents an essential feature of affective processing (McEvoy et al., 2013). Worry represents a chain of negative thoughts about potential catastrophic outcomes in the future (Borkovec et al., 1983). While most conceptualizations incorporate worry as a facet of anxiety (Davey et al., 1992), worry and the related process of rumination also present across a spectrum of emotional problems, including anxiety and depression in both their subsyndromal expression and as major clinical disorders (Muris et al., 2005; Hong, 2007; Hsu et al., 2015).

Impaired “attentional control” is a key component of worry and rumination (Armstrong et al., 2011; Hsu et al., 2015). Attentional control refers to the efficient use of attention resources during goal-directed tasks, namely inhibitory control or attention shifting (Derakshan and Eysenck, 2009). Worry can impair cognitive control. It lowers efficient processing of goal-directed information, which can impair inhibitory control and shifting on tasks (Derakshan and Eysenck, 2009), or what are commonly referred to as cognitive control deficits. Another pathway implicated in studies of worry or rumination is impaired attentional control predisposing to perseverative negative thinking (Armstrong et al., 2011; Fox et al., 2015; Hsu et al., 2015; Macatee et al., 2016). Several lines of evidence suggest that worry is associated with impaired cognitive control. For example, neuroimaging studies link dysregulated neural networks relevant to cognitive control to worry. Among middle-aged and older adults, worry symptom severity, but not global anxiety, uniquely predicted abnormal neural connectivity of the salience detection network (Andreescu et al., 2015). This network of brain structures (amygdala, parts of the insula, anterior cingulate cortex, thalamus) monitors relevance and importance of stimuli (Menon and Uddin, 2010). This network has been implicated in both cognitive control performance and more generally in emotion regulation (McTeague et al., 2017). Dysregulated salience monitoring could explain problems with attentional control, given that misattributing emotional salience to ordinary stimuli could cause difficulty in disengaging (i.e., shifting and focusing) attention away from mundane stimuli (Etkin et al., 2006). Altogether, these investigations provide a neurobiological basis for cognitive control deficits being associated with elevated worry, suggestive of worry as both a cognitive and affective symptom.

The second line of evidence that worry has a detrimental effect on cognitive control and memory processes comes from geriatric populations, where cognitive control is often decreased (i.e., complex cognitive processes including working memory and inhibitory control; Drag and Bieliauskas, 2010). Two studies in community-residing older individuals support this view. In one, “mildly elevated” worry symptoms at baseline were associated with lower verbal memory recall on a word list task and predicted a clinically relevant decline in visual memory performance two-years later (Pietrzak et al., 2012). The authors postulated that “speed-dependent processing, learning, and higher-level executive functions such as shifting, inhibition, and reasoning” (p. 7) are deleteriously affected by worry. In the second study, higher self-reported worry correlated with poorer set-shifting, but not with other cognitive control or memory measures (Yochim et al., 2013).

Among older adults, anxiety is related to poorer cognitive control (Beaudreau and O'Hara, 2009; Yochim et al., 2013). Late-life depressive disorders have broader associations, including memory deficits and cognitive control deficits (Butters et al., 2004). Yet, researchers (Yochim et al., 2013) found late-life depressive symptoms were associated with poor delayed verbal memory but not cognitive control. Other studies in older adults find no significant association between elevated anxiety and cognitive control (de Bruijn et al., 2014; Delphin-Combe et al., 2016) or between memory and elevated depressive symptoms (Beaudreau and O'Hara, 2009). Further, elevated anxiety in older adults also has been associated with lower memory (Delphin-Combe et al., 2016), albeit possibly due to cognitive control deficiency (Yochim et al., 2013). Depressive symptoms and disorders associated with cognitive control dysfunction may be due to information processing and working memory deficits (Bhalla and Butters, 2011). Worry as a modulator of affective symptom expression on cognition function has been inadequately interrogated. Inconsistencies regarding past findings of associations of anxiety and depression with cognitive processing could be driven by the degree of participant worry, which is rarely measured or considered.

Another limitation is that prior investigations often focus solely on severe affective symptoms. Capturing the full range of symptoms, as inspired by the NIH Research Domain Criteria (RDoC; Cuthbert, 2014), may provide finer-grained information on inter-relationships among worry, anxiety and depression on neurocognitive processing in later life. The current investigation focuses on milder affective symptoms experienced by community-residing older adults, in whom investigations routinely describe cognitive issues in the context of elevated affective symptoms (Beaudreau and O'Hara, 2009; Weisenbach et al., 2012; Yochim et al., 2013). Although, worry may adversely impact cognitive control processes, it is unclear how much worry accounts for negative associations between the dimensional symptoms of late-life anxiety and depression with cognitive control and verbal delayed memory processes, and more precisely, if worry serves to exacerbate these associations.

Here we predicted that worry would account for associations between symptoms of anxiety and depression with cognitive control and delayed verbal memory processes in community-dwelling older adults. We expected that worry would drive poorer cognitive performance, directing and intensifying associations of affective symptoms on specific cognitive abilities for two reasons. First, poorer attentional control is associated with worry and has been proposed as a potential risk factor for worry (Macatee et al., 2016). Second, findings of community-dwelling older adults with milder symptoms implicate greater worry as potentially detrimental to cognitive control and delayed memory processes (Pietrzak et al., 2012; Yochim et al., 2013). We hypothesized that increased worry symptoms would negatively impact inhibitory control, a cognitive control ability that has been uniquely associated with anxiety in prior investigations (Beaudreau and O'Hara, 2009), and that increased levels of worry would further exacerbate the negative association of anxiety symptoms with inhibitory control processes.

Similarly, based on work linking depression with poor delayed verbal recall (Bhalla et al., 2006; Weisenbach et al., 2012; Yochim et al., 2013), we hypothesized that increased levels of worry would negatively impact delayed verbal memory and exacerbate the negative relationship between increased depressive symptoms and poorer delayed verbal memory. In secondary analyses, we examined additional cognitive control abilities (working memory, information processing, category fluency) and visual memory recall to determine if the effects of worry are broadly associated with cognitive control and memory processes, or if associations were specific to inhibitory control and verbal memory.

In sum, we aimed to determine whether worry accounts for, or exacerbates, known negative associations between the symptoms of late-life anxiety and depression with cognitive control and delayed verbal memory processes in community-dwelling older adults.

## Materials and methods

### Participants

Community-dwelling older adults (*n* = 122) aged 65 and older, who passed a brief cognitive screen (Katzman et al., 1983) participated in the study. Study exclusions included (a) not passing the cognitive screen (≥6 errors on the Blessed Object Memory and Concentration Test; Katzman et al., 1983), (b) a previous diagnosis of dementia (any cause, including but not limited to Alzheimer's disease, vascular or stroke, or Parkinson's), (c) cognitive testing indicative of mild cognitive impairment or dementia, (d) presence of psychotic symptoms, or (e) restricted English fluency sufficient to interfere with testing. Recruitment occurred through multiple outlets to attract diverse older adults in the community: an institutional newsletter distributed to older adults in the community, flyers posted in the medical center including primary care clinic waiting rooms and elevators, internet advertisements, and word-of-mouth to local research clinics providing services to older adults. We excluded three participants because of active psychotic symptoms (*n* = 1), anomalous cognitive performance and social behavior despite passing the screen and denying psychiatric symptoms (*n* = 1), or unusual cognitive performance due to English fluency issues (*n* = 1). The final sample was 119.

### Study design

The study design focused on cross-sectional data from an observational investigation conducted from 2010 to 2012 approved by the Stanford University IRB and in adherence to the ethical guidelines of the Declaration of Helskinki. Participants orally consented to the phone screen and provided written informed consent prior to completing in-person study assessments, which consisted of testing with a psychologist (SB) or a trained bachelor's or master's level research assistant. Participants completed demographic and health questions, psychiatric interviews (i.e., Structured Clinical Interview for the DSM-IV; SCID-IV; Spitzer et al., 2002), mood and anxiety questionnaires, and a cognitive battery. SCID-IV interviews achieved excellent interrater reliability (Kappa = 0.87). Enrollees received $50 compensation for 5 h of baseline testing.

### Measures

Demographic information, medical history, and perceived health in relation to peers (excellent “1” to poor “4”) were assessed by questionnaire. Self-rated health has empirical support a robust indicator of the presence of single chronic medical conditions, including stroke, or cardiovascular disease (Mavaddat et al., 2014). The assessment included several self-report instruments of affective symptom severity validated for use with older adults. The Penn State Worry Questionnaire (PSWQ) measured worry and has good internal consistency (coefficient alpha = 0.83) in older adults (Meyer et al., 1990; Stanley et al., 2001). The Beck Anxiety Inventory (BAI), which has excellent internal consistency in older adults (Cronbach's alpha = 0.93), measured anxious arousal (Beck et al., 1988; Wetherell and Arean, 1997). Lastly, the Beck Depression Inventory-II (BDI-II), also a valid and internally consistent measure in older adults (Cronbach's alpha = 0.86), assessed depressive symptoms (Beck et al., 1996; Segal et al., 2008).

Primary neurocognitive measures included delayed word recall (Rey Auditory Verbal Learning Test, RAVLT; Lezak, 1983) and inhibitory control on a Stroop interference task (Color-Word Interference Test, CWIT, Condition 3 Inhibition Trial; Delis et al., 2001). Inhibitory control processes on the Stroop CWIT were estimated with speed of processing (time to complete in seconds) and accuracy maintaining the goal or instances of set loss (errors). Two error types were assessed; uncorrected errors, in which there is a lack of awareness that a set loss occurred, and self-corrected errors, in which there is a spontaneous correction of a set loss. Faster speed and fewer set losses are metrics of good inhibitory control processes. Secondary neurocognitive tests included delayed recall of geometric figures (Visual Reproduction test; Wechsler, 2008); category fluency for animal words and boys names (Delis et al., 2001); attention and working memory (Digit Span; Wechsler, 2008), and processing speed and shifting attention (Digit Symbol Coding; Wechsler, 2008).

### Data analyses

To test primary hypotheses, we conducted four multiple linear regression models, one for each cognitive measure: three indices of inhibitory control (Stroop CWIT) and delayed verbal memory (RAVLT). Independent variables entered into the model included the main effects, second order interactions, and third order interactions among PSWQ, BAI, and BDI-II total scores. All models were adjusted for age, education, sex, and perceived health because these variables have been reported to be associated with late-life cognition. The inclusion of perceived health has been found to be a good proxy measure of single chronic medical conditions such as stroke or cardiovascular disease with neurobiological links to late-life affective dysregulation (e.g., Popa-Wagner et al., 2014). Independent variables were centered as deviations from the median, except for sex which was coded as 0.5 and −0.5 (Kraemer and Blasey, 2004). The *p*-value for the primary hypotheses was *p* < 0.05 and Bonferroni corrected for secondary analyses using *p* < 0.01 to test models examining the other five cognitive control and memory tests.

## Results

### Descriptive statistics

Participant baseline characteristics are summarized in Table 1. As expected, participants reported symptoms indicative of minimal anxiety, worry and depression on average. Nonetheless, 35.3% (*n* = 42) reported anxiety, worry or depressive symptoms above clinical cut-offs (report of at least mild symptoms on the BAI or BDI-II or >45 on the PSWQ; Behar et al., 2003). Further, 18.5% (*n* = 22) of the sample met criteria for a current SCID-IV psychiatric diagnosis (Spitzer et al., 2002), suggesting that the sample captured a full spectrum of symptoms from normal to abnormal using a dimensional approach consistent with the RDoC framework (Cuthbert, 2014).

**Table 1:** Baseline descriptives for sample characteristics, psychiatric measures, and cognitive tests.

	**Mean/% (*n*)**	***SD***	**Min-Max**
**SAMPLE CHARACTERISTICS (*****n*** **= 119)**			
Age	74.16	6.82	65–91
Education§	16.82	2.41	10-20+
Sex (female)	56.3 (67)		
Marital Status			
Married	52.1 (62)		
Divorced/Separated	23.5 (28)		
Widowed	13.4 (16)		
Single	10.9 (13)		
Ethnicity/Race			
White, Non-Hispanic	90.8 (108)		
Asian American	5.9 (7)		
African American	2.5 (3)		
Latino	0.8 (1)		
Native Language			
English	105 (88.2)		
Other Language	14 (11.8)		
**PSYCHIATRIC MEASURES**			
Total Score			
BAI	3.74	5.21	0–29
BDI-II	5.56	7.34	0–41
PSWQ	37.66	12.92	16–76
Clinical Cut-Score
BAI (mild to severe)	11 (9.2)		
BDI-II (mild to severe)	16 (13.5)		
PSWQ (> 45)	30 (25.2)		
**COGNITIVE CONTROL**			
CWIT^*^			
Time (secs)	65.54	16.37	38–121
Self-corrected errors	0.95	1.43	0–8
Uncorrected errors	0.44	1.67	0–17
Digit Span			
Forward	10.22	2.42	5–16
Backwards	8.96	2.47	2–16
Digit-Symbol Coding	59.32	13.77	28–91
Category Fluency	39.81	8.91	15–58
**MEMORY RECALL**			
RAVLT-Delay^*^	9.28	3.28	1–15
Visual Reproduction-Delay	24.32	8.45	0–43

PSWQ, Penn State Worry Questionnaire; BDI-II, Beck Depression Inventory—II; BAI, Beck Anxiety Inventory; CWIT, D-KEFS Color-Word Interference Test Condition 3-Interference Trial; RAVLT-Delay, Rey Auditory Verbal Learning Test Long Delay Free Recall; A clinical cut-score of 45 on the PSWQ was selected based on a previously published study (Behar et al., 2003). Unit of measurement for all neuropsychological tests was number of items correct or errors when noted, except for CWIT Time, which is test completion time in seconds. §Education above 20 years coded as 20+ years;

* *Cognitive tests examined in the primary analyses using p < 0.05*.

### Primary analyses

#### Inhibitory control

**Figure 1:**
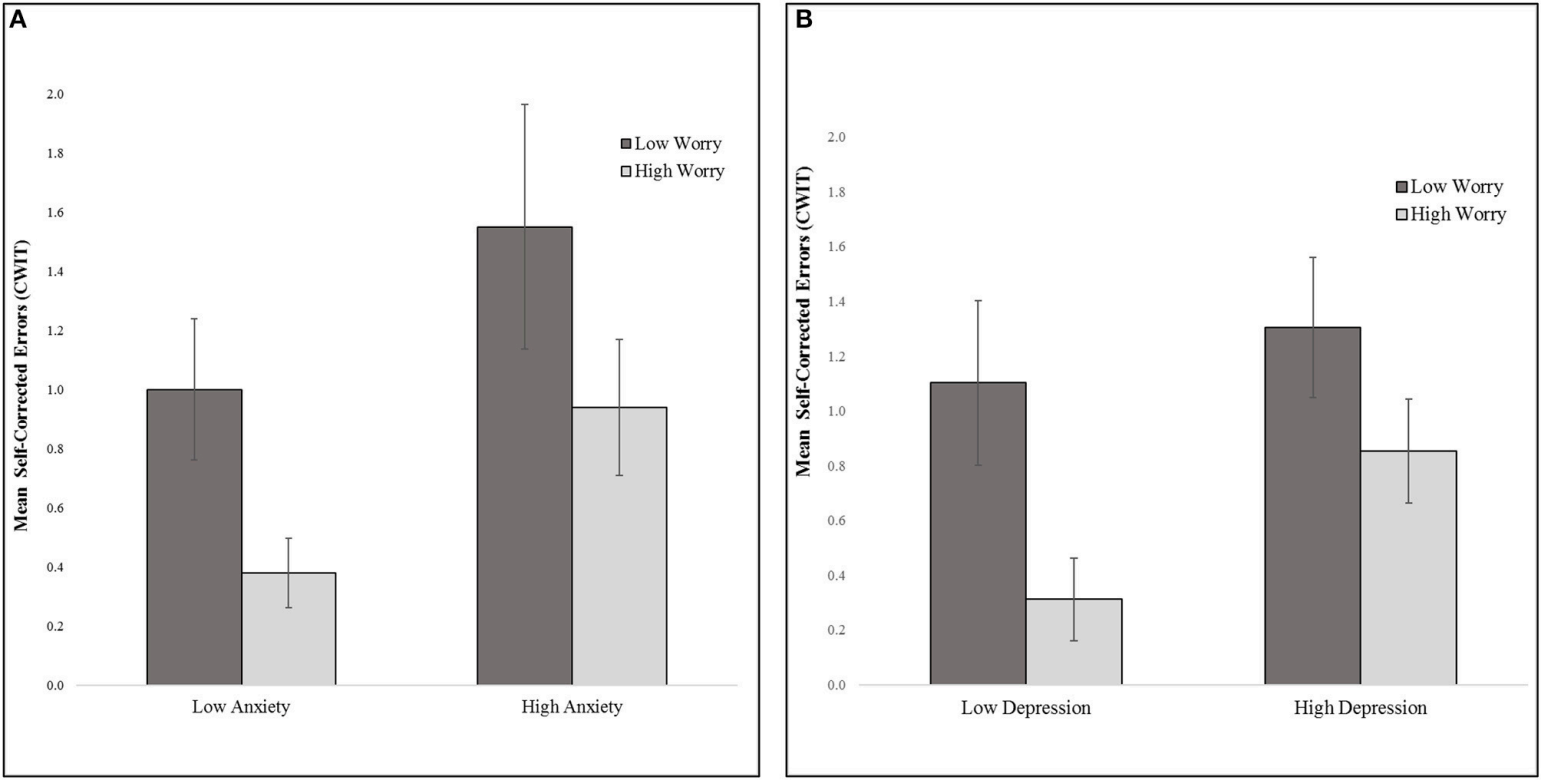
Inhibitory control (Mean CWIT self-corrected errors) **(A)** worry by anxiety (median split on worry and anxiety symptom total scores) **(B)**. worry by depression (median split on worry and depressive symptom total scores). CWIT, D-KEFS Color-Word Interference Test condition 3-interference trial.

Overall, main effects for affective symptoms were not significant, but the two-way interactions among affective symptoms were, accounting for 9.5% of unique variance in Stroop CWIT self-corrected errors (Table 2). Specifically, worry interacted with anxiety symptoms to predict Stroop CWIT self-corrected errors, *t*_(108)_ = 3.05, *p* = 0.003, whereby less worry and greater severity of anxiety were associated with more CWIT self-corrected errors (a negative indicator of inhibitory control processes). More worry attenuated this association between greater severity of anxiety and self-corrected errors (see Figure 1A).

**Table 2:** Multivariate model summary of baseline analyses for affective symptoms as predictors of inhibitory control and delayed verbal memory recall.

**Inhibitory control (CWIT)**	**R square change**	**F change**	***P*-value**
**SELF-CORRECTED ERRORS**			
1	0.06	1.83	0.13
2	0.05	2.13	0.10
**3**	**0.10**	**4.32**	**0.006**
4	0.00	0.57	0.45
**UNCORRECTED ERRORS**			
1	0.05	1.42	0.23
2	0.01	0.19	0.90
3	0.01	0.20	0.90
4	0.01	1.23	0.27
**TIME TO COMPLETE**			
1	0.08	2.45	0.05
2	0.00	0.10	0.96
3	0.04	1.67	0.18
4	0.00	0.06	0.81
**DELAYED VERBAL MEMORY (RAVLT-7)**			
**1**	**0.26**	**10.23**	**0.0001**
2	0.00	0.12	0.95
**3**	**0.08**	**4.10**	**0.008**
4	0.00	0.00	0.95

*CWIT, D-KEFS Color-Word Interference Test Condition 3-Interference Trial; RAVLT-7, Rey Auditory Verbal Learning Test-Delay Trial 7; Multiple Linear Regression step 1, demographics (age, sex, education in years) and a health rating in comparison to one's peers (dfs = 4, 114); 2, main effects (dfs = 3, 111), 3, two way interactions (dfs = 3, 108), and 4, three-way interactions (dfs = 1, 107) of BAI total score, BDI-II total score, PSWQ total scores. Bold values indicates statistically significant (p < 0.05)*.

Further, worry interacted with depressive symptoms to predict Stroop CWIT self-corrected errors, *t*_(108)_ = −2.16, *p* = 0.033. Specifically, less worry and greater depressive symptom severity was associated with more Stroop CWIT self-corrected errors (see Figure 1B).

Inhibitory control as measured by time to complete or uncorrected errors on the Stroop CWIT was not associated with anxiety, depressive, or worry symptoms (Table 2).

#### Delayed verbal memory

**Figure 2:**
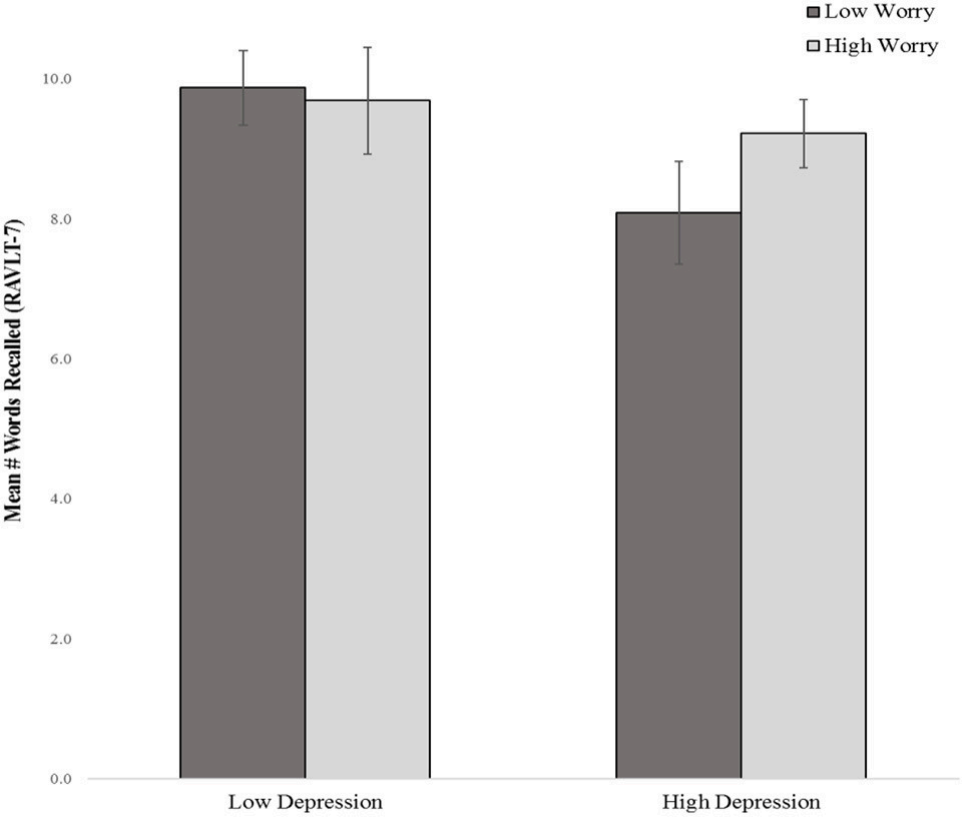
Delayed verbal recall by depressive and worry symptom severity (median split) RAVLT-7, Rey Auditory Verbal Learning Test- Delay Trial 7.

The main effects for affective symptoms were not significant in the model. However, two-way interactions between depressive, and worry symptoms were, accounting for an additional 7.5% of the variance in delayed verbal memory after adjusting for demographics, perceived health, and the main effects of symptoms. *T*-tests revealed that worry interacted with depressive symptoms in association with delayed verbal memory, *t*_(108)_ = 2.66, *p* = 0.009. Specifically, individuals reporting high depressive symptoms but low worry symptoms, or high worry symptoms but low depressive symptoms, recalled fewer words as compared to those with no symptoms or high severity of both symptoms (Figure 2). No other symptoms were significant predictors of memory in the model (see Table 2).

### Secondary analyses

Anxiety, depressive and worry symptoms did not emerge as significant main effects or interactions in any of other models of cognitive control (attention and working memory, information processing and processing speed, category fluency) or visual memory.

## Discussion

Our study provides compelling support for worry severity impacting the relationship of anxiety and depression to specific neurocognitive processes in community-residing older adults. Counter to prediction, however, worry did not exacerbate negative associations between affective symptoms and cognitive processing. Instead, worry appeared to attenuate the negative effects of elevated depressive and anxiety symptoms on specific late-life inhibitory processes and to attenuate the negative effects of elevated depressive symptoms on delayed verbal memory processes. Specifically, with regard to inhibitory processes, high worry was associated with fewer spontaneously corrected errors on a Stroop task in the presence of high anxiety and high depression. Critically, this tendency for high worriers to make fewer spontaneous corrections of errors in the context of high anxiety or depression was not the result of making more uncorrected errors or having a slowed speed of processing on the inhibitory task. The specificity of this result is further emphasized by our secondary analyses in which there were no significant main effects or interactions of worry with other cognitive control processes.

This striking neurocognitive association implicates fewer careless errors and greater precision in the inhibitory processes of older adult worriers with high anxiety or high depression than among those reporting low worry. This could be explained in part by a personality trait of high worriers such as perfectionism, and more specifically a brand of perfectionism characterized by an intrinsic motivation for error-free performance (Stahl et al., 2015). Certainly, a recent investigation found just that—adults reporting high intrinsic perfectionism performed with greater accuracy following an error on a trial as compared with low intrinsic perfectionists (Stahl et al., 2015). This tendency to focus on personal goals, as with intrinsic perfectionists who might be more likely to also engage in high worry, could be one explanation for associations between high worry and fewer self-corrected errors. Though perfectionism was not measured in this study, future studies could further delineate the role of intrinsic perfectionism with regard to this neurocognitive finding.

At the least, these findings are suggestive of fewer self-corrected errors during inhibitory processes as a neurocognitive phenotype of late-life worry with high anxiety or high depression. This neurocognitive phenotype is potentially applicable across the lifespan given another study that also found the same association of fewer self-corrected errors, but no differences in time to complete or uncorrected errors, on the Stroop task in young women reporting higher worry (Janowski et al., 2009).

Delayed verbal memory processes benefited from high worry in older adults with high depression relative to those with high levels of depression but low levels of worry. In other words, older adults with high depression recalled more words if they also had high levels of worry than if they had low levels of worry. Strikingly, the worry severity no longer seemed to impact delayed verbal memory when depression was low. Again, this finding was specific to verbal memory with no significant finding for delayed visual memory. This could be possibly due to verbal memory requiring more cognitive effort to free recall words than is required by recall of geometric figures, the overlap in cognitive processes between the verbal nature of worry and verbal memory, or potentially benefitting from increased motivation due to worry-related personality attributes, such as perfection. The specificity of this result implies different neurocognitive phenotypes of delayed verbal memory in those with high depression depending on their level of worry.

The specificity of our neurocognitive findings are not readily explained by the attentional control hypothesis. Neurocognitive processing occurred in a naturalistic context, in which no negative distractors were presented. Future studies comparing the neurocognitive processes of community-dwelling older adults with milder worry, anxiety, and depressive symptoms under conditions of no distractors vs. negatively valenced distractors would be equipped to observe if these neurocognitive processes change in a “threat” context—either increasing the magnitude or direction of our findings. Further, worry above or below a certain threshold (pathological vs. non-pathological) could differentially influence associations between affective symptoms and neurocognitive processes.

Our current findings have implications for the NIMH RDoC framework and continued development of subgroup classification of affective symptoms based on associated neurocognitive profiles. The dominant goal of RDoC is to improve classification of mental disorders using a dimensional approach that incorporates multiple levels of analysis from basic neurobiology to behavior (Cuthbert, 2014). Most relevant to late-life affective symptoms and their associated neurocognitive processes are proposed functional systems of negative affect (Negative Valence systems) and cognitive functioning (Cognitive systems). Though not currently subsumed under the negative valence dimension, a perseverative thought process, such as worry, may be an important potential candidate for future inclusion, as a specific, cross-cutting functional mechanism of emotion dysregulation (Ruscio et al., 2011). By harnessing what is known about perseverative negative thought, we might reach greater understanding of the heterogeneity of anxiety and depressive disorders, including mixed and independent phenotypes (Ruscio et al., 2011). Our nuanced approach of neurocognitive phenotypes lends further support to consideration of worry and other preservative thought processes as a functional category for inclusion in the RDoC.

Continued development of this work could also impact treatment development, particularly for late-life psychiatric issues. Worry symptoms are notoriously difficult to treat and have been cited as one of the main reasons that the treatment of generalized anxiety disorder yields such disappointing effect sizes in younger and older adults (Gould et al., 2012). These patients may in fact suffer a more difficult to treat type of anxiety or depression.

The cross-sectional nature of our investigation cannot speak to the direction of association and our findings may not generalize to older adult samples with less than a college education or with significant cognitive impairment. Longitudinal studies that include worry and its interactions with anxiety and depressive symptom severity would be poised to determine if these symptom expressions alter the trajectory of cognitive control and memory processing in older adults. Of further interest, symptom expression tied to neurobiological markers, such as neuroinflammation, could elucidate the pathways leading to specific cognitive processing phenotypes in older adults (Sandu et al., 2015). In addition to community samples, testing of these models in chronically medically ill elders would be illuminating.

Future studies could examine whether self-corrected errors during inhibitory control and delayed verbal memory processes predict psychiatric subgroups based on change in dimensional symptom severity of anxiety, depressive and worry symptoms. Recent work has uncovered a depressive phenotype of cognitive impairment and decline (Johnson et al., 2013). Future studies using item-level analyses of symptom measures could lead to the development of an affective phenotype of cognitive function in late life (Johnson et al., 2013). Also consistent with the RDoC framework (Cuthbert, 2014), discovery of genetic moderators of cognition or affective symptoms could further refine knowledge about these cognitive profiles of worry and other affective symptoms to develop endophenotypic profiles that link cognitive “traits” with psychiatric symptom characteristics. Further development of this work oriented toward more fine-grained phenotyping of psychiatric subgroups and the application of this knowledge to compare clinical outcomes in these groups remains an exciting, relatively unexplored direction that could enhance late-life treatment outcomes studies of the future.

## Author contributions

SB designed study and obtained the funding in support of the study; RO provided ongoing scientific consultation on the design of the study and throughout its conduct; SB analyzed the study results; All authors wrote and provided substantive intellectual feedback during the writing of the manuscript.

### Conflict of interest statement

The authors declare that the research was conducted in the absence of any commercial or financial relationships that could be construed as a potential conflict of interest.
